# A Novel 4H-SiC SGT MOSFET with Improved P+ Shielding Region and Integrated Schottky Barrier Diode

**DOI:** 10.3390/mi15070933

**Published:** 2024-07-22

**Authors:** Xiaobo Cao, Jing Liu, Yingnan An, Xing Ren, Zhonggang Yin

**Affiliations:** 1 Department of Electrical Engineering, Xi’an University of Technology, Xi’an 710048, China; 1231910007@stu.xaut.edu.cn (X.C.); zhgyin@xaut.edu.cn (Z.Y.); 2 Department of Electronic Engineering, Xi’an University of Technology, Xi’an 710048, China; 2220321291@stu.xaut.edu.cn (Y.A.); 2220320020@stu.xaut.edu.cn (X.R.)

**Keywords:** SiC SGT MOSFET, P+ shielding region (PSR), breakdown voltage, on-resistance, reverse recovery

## Abstract

A silicon carbide (SiC) SGT MOSFET featuring a “一”-shaped P+ shielding region (PSR), named SPDT-MOS, is proposed in this article. The improved PSR is introduced as a replacement for the source trench to enhance the forward performance of the device. Its improvement consists of two parts. One is to optimize the electric field distribution of the device, and the other is to expand the current conduction path. Based on the improved PSR and grounded split gate (SG), the device remarkably improves the conduction characteristics, gate oxide reliability, and frequency response. Moreover, the integrated sidewall Schottky barrier diode (SBD) prevents the inherent body diode from being activated and improves the reverse recovery characteristics. As a result, the gate-drain capacitance, gate charge, and reverse recovery charge (*Q_rr_*) of the SPDT-MOS are 81.2%, 41.2%, and 90.71% lower than those of the DTMOS, respectively. Compared to the double shielding (DS-MOS), the SPDT-MOS exhibits a 20% reduction in on-resistance and an 8.1% increase in breakdown voltage.

## 1. Introduction

Nowadays, wide-bandgap devices are generally used in high-voltage and high-power applications. Silicon carbide, as one of the most promising materials in wide-bandgap semiconductors, has excellent characteristics and can be used to make devices with superior performance at high temperature, high power, high reliability, and high speed [1,2,3]. SiC MOSFETs have a smaller chip area, much higher switching frequency, and a smaller on-state resistance (*R_on_*) than those of the Silicon Insulated Gate Bipolar Transistor (IGBT), and have wide potential applications in areas such as electric vehicles, photovoltaic inverters, uninterruptible power supplies, and energy distribution networks [4,5,6].

**Figure 1:**
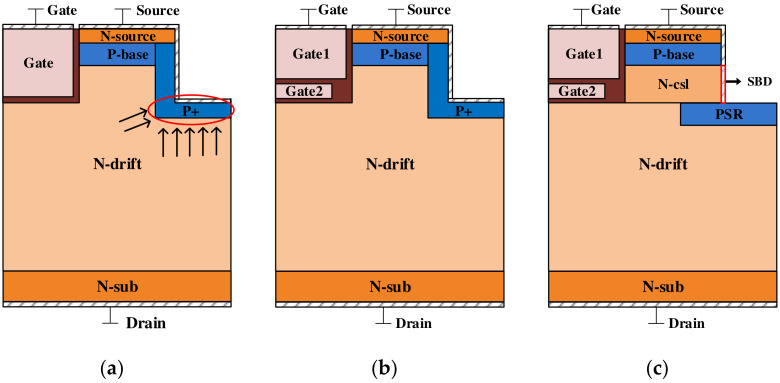
Cross-sectional view of a (**a**) DT-MOSFET, (**b**) DS-MOSFET, and (**c**) SPDT-MOSFET.

Compared with planar-gate DMOS devices, trench-gate MOS devices eliminate the JFET region and the channel density can be made larger by using a smaller cell pith with a lower *R_on_* and a higher power density [7]. However, when trench MOS devices operate in blocking mode, the exposed edge of Poly-Si increases the electric field in the gate oxide, which threatens the device’s long-term reliability [8,9,10]. In order to address these issues, SiC trench MOSFETs with a P-type shield layer under the trench bottom and a double-trench structure have been proposed [11,12]. A double-trench structure with a p-type region—which is deeper than the bottom of the gate trench—at the bottom of the source trench has been suggested [13]. Figure 1a shows the schematic cross-section of a 4H-SiC trench MOSFET with a double-trench (DT-MOSFET). The source trench effectively alleviates the peak electric field at the corner of the trench oxide and improves the breakdown voltages (BVs) [14]. However, the PN junction depletion region formed by the L-type source groove and the N-type drift layer in the device structure can lead to certain challenges. One of these challenges is that the depletion region narrows the current path, which increases the on-resistance of the device. Additionally, the overlap area between the gate and the drain is large in this structure, leading to large gate-to-drain capacitance (*C_GD_*). The *C_GD_* can negatively affect the switching speed and overall performance of the device. It can cause delays in turning the device on and off, resulting in increased power losses and reduced efficiency [15]. To address these issues, researchers have proposed the double split-gate SiC MOSFET (DS-MOSFET) with a shielded gate design, which helps to reduce the *C_GD_* and improve the device’s switching characteristics and efficiency [16].

The DS-MOSFET with a grounded split-gate and source trench is shown in Figure 1b. The SG located below the gate trench is connected to the source electrode and acts as a shielding region between the gate and drain, transforming part of the *C_GD_* into the drain-to-source capacitance (*C_DS_*) and gate-to-source capacitance (*C_GS_*) in series, reducing the *C_GD_* of the device, and improving the switching characteristics [17]. The source trench sidewall of the DT-MOS forms a depletion region with the drift region, leading to reduction in the device’s conduction characteristics. On the other hand, the DS-MOS improves the switching characteristics of the DT-MOS by introducing a split gate. But this further reduces the conduction area at the bottom of the trench, significantly deteriorating the device’s conduction characteristics. Importantly, the introduction of SG in the DS-MOS causes a significant concentration of electric field lines at the bottom of the gate oxide in the forward blocking state. This results in the maximum electric field in the gate oxide exceeding the safe threshold, leading to compromised device reliability. In this paper, we conducted research on achieving a low *R_on_* and high reliability in these devices.

This paper proposes the introduction of an improved P+ shielding region (PSR) and SBD in SGT MOSFETs (referred to as the SPDT-MOSFET), which achieves a high Baliga figure of merit (BFOM) and superior switching performance. The proposed SiC MOSFET introduces the PSR with an improved shape, which expands the conductive path of the source trench sidewall and minimizes the coupling area between the source-gate trench. Additionally, the improved PSR layer, with its well-designed configuration, can effectively alleviate the issue of excessive electric field at the bottom gate oxide layer caused by the introduction of a split gate in the DS-MOS. The SPDT-MOSFET integrates an SBD on the sidewall of the source trench, which effectively reduces the cell size and avoids bipolar degradation of the device, thus optimizing the overall performance of the device [18,19,20,21,22,23]. In the blocking state, the PN junction formed by the PSR and N-drift layer can withstand high voltages, which improves the reliability of the gate oxide and reduces the surface electric field intensity of the Schottky junction. As a result, the proposed structure exhibits a lower leakage current and improved reliability.

## 2. Device Structure and Characteristics

The SPDT-MOSFET structure is shown in Figure 1c. In the proposed SiC MOSFET, the “一”-shaped PSR is introduced to attract electric field lines, thereby reducing the gate oxide electric field intensity. The PSR forms an auxiliary depleted drift region in conjunction with the N-drift layer. In comparison to the structures delineated in Figure 1a,b, the introduction of the “一”-shaped PSR effectively reduces the lateral depletion region width and expands the current conduction path of the CSL within the SPDT-MOSFET structure. Consequently, the *R_on_* of the SPDT-MOSFET is slightly lower than that of the conventional DT-MOSFET.

In the blocking state, the PN junction formed by the PSR and the N-drift layer beneficially withstands a proportion of the blocking voltage. This effectively alleviates the peak electric field at the corner of the trench gate oxide. Furthermore, the Schottky contacts are formed on the sidewall of the source trenches of the SPDT-MOSFET, with its SBD electrode deliberately tied to the source electrode, serving to suppress the activation of the device’s intrinsic diode during the commutation phase [7]. Consequently, this deliberate action effectively circumvents detrimental bipolar degradation effects while simultaneously elevating the device’s reverse recovery characteristics [24,25].

**Figure 2:**
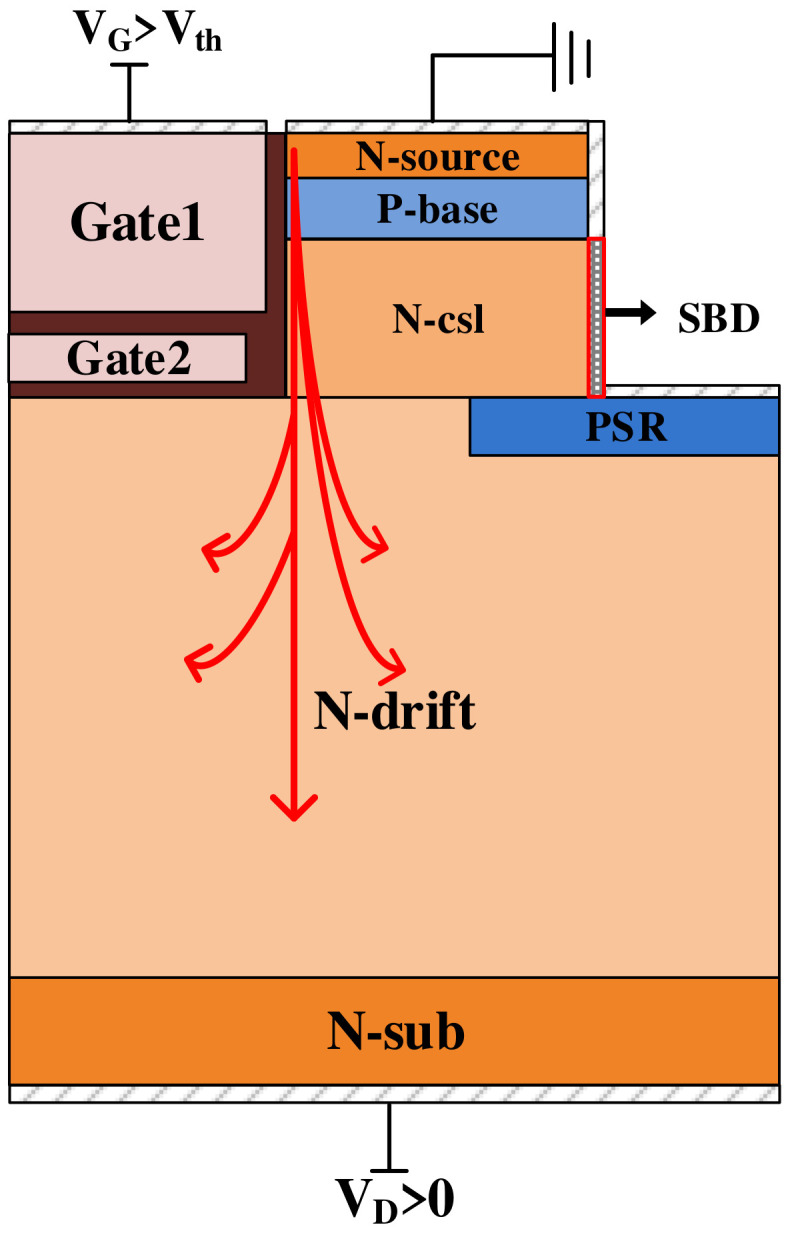
Current path in the forward conduction state of the proposed SiC MOSFET.

Figure 2 illustrates the forward conduction current transport mechanism of the SPDT-MOSFET. When *V_GS_* > *V_th_*, the SPDT-MOSFET channel becomes active. At the same time, electrons traverse the P-base region, the *N_CSL_* layer, and the drift region, proceeding from the source terminal and ultimately reaching the drain terminal. The improved PSR and *N_CSL_* layer introduced in this paper also significantly reduces *R_on_* and expands the current conduction pathway in the *N_CSL_* layer in the proposed SiC MOSFET.

**Figure 3:**
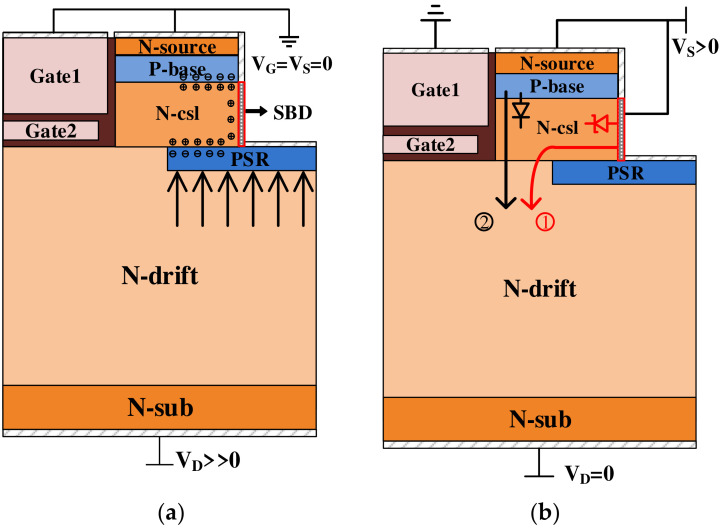
Schematic view of (**a**) the withstand voltage mechanism in the forward blocking state; (**b**) the SBD and body diode working mechanism of the proposed SiC MOSFET.

When *V_GS_* = 0 V and *V_DS_* >> 0, the MOSFET operates in the forward blocking state, while the Schottky diode is in the reverse bias state. For Schottky diodes integrated in SPDT-MOSFETs, as the electric field at the Schottky’s contact surface increases, the barrier lowering effect and the tunneling effect of the Schottky contact cause a decrease in the barrier height, resulting in an increase in leakage current. In this situation, the introduction of the improved PSR layer creates numerous acceptor centers. These acceptor centers combined with the P-base region, causing the concentration of electric field lines from the drift region onto the improved PSR. This effectively decreases the electric field at the Schottky contact and alleviates the peak electric field at the corner of the trench oxide. The improved PSR and the P-base region jointly produce the reverse voltage blocking, as shown in Figure 3a.

Figure 3b shows the schematic diagrams of the SBD and body diodes of the SPDT-MOSFET. The Schottky metal of the source trench sidewall and N-drift form the Schottky diode, and the P-base and N-drift/N+-drain form the body diode. Under reverse conduction, the different turn-on voltage of the two diodes causes the device to have double conductive modes. As the reverse voltage increases, the SBD turns on first, allowing current to flow through the Schottky metal and N-drift layer. Subsequently, when the reverse voltage surpasses the turn-on voltage of the P-i-N diode, it also begins to conduct current because the SBD exhibits a lower turn-on voltage compared to the P-i-N diode. At this time, the integrated SBD and the body diode are connected in parallel at the source and drain terminals. When the source-drain voltage is constant, more current flows into the drain end through the SBD and the conduction of the body diode is suppressed, which further reduces switching losses [19]. Consequently, operating the device in a unipolar conduction mode effectively prevents bipolar degradation, enhancing the device’s reliability and reverse recovery characteristics.

## 3. Results and Discussion

In this study, Sentaurus TCAD is used to perform the device simulations and the mixed-mode simulations [26]. The design takes into account several fundamental models, including Shockley–Read–Hall recombination, Auger recombination, Okuto–Crowell collision ionization, barrier lowering, anisotropic material properties, and more [14]. The utilized models and key parameters have been simulated and fitted to closely match the testing curve of the 1200 V 22 mΩ DT MOSFET (SCT3022KL) device. This article compares and analyzes the characteristics of the SPDT-MOSFET, DS-MOSFET, and DT-MOSFET, highlighting the advantages of the SPDT-MOSFET, such as its reduced *C_GD_*, lower reverse conduction voltage, and enhanced switching speed. The key parameters of these devices are shown in Table 1.

**Figure 4:**
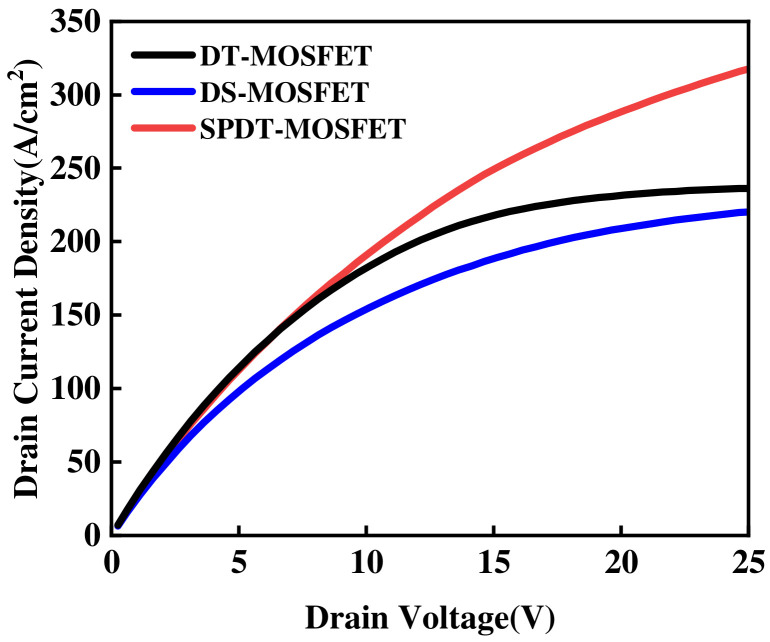
I–V characteristics in first-quadrant operation for three devices.

Figure 4 illustrates the forward conduction I-V characteristics of the three devices under a gate-source voltage (*V_GS_*) of 15 V and a drain-source voltage (*V_DS_*) of 50 V. The graph clearly demonstrates that the conduction performance of the DS-MOSFET devices shows a slight degradation in comparison to that of the DT-MOSFET, whereas the SPDT-MOSFET devices exhibit significantly enhanced conduction characteristics that surpass both devices. When the *V_DS_* is at 1 V, the comparative *R_on_* of the SPDT-MOSFET, DS-MOSFET, and DT-MOSFET devices are measured to be 2.4 mΩ·cm^2^, 4.2 mΩ·cm^2^, and 2.8 mΩ·cm^2^, respectively. This reveals a significant decrease of 42.8% and 14.2% in *R_on_* compared to the DS-MOSFET and DT-MOSFET, respectively. In the proposed SiC MOSFET, the “一”-shaped PSR is introduced while retaining the split gate, and the issues of a narrowed current path and increased *R_on_* due to the split gate are addressed. Moreover, the inclusion of an *N_CSL_* layer further enhances the device’s conduction capacity, ensuring an improved overall performance.

**Figure 5:**
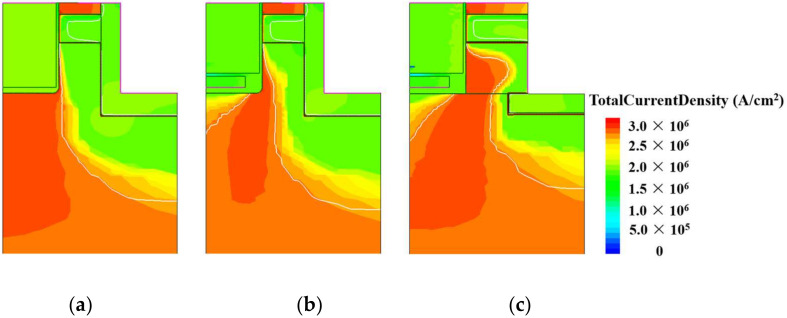
The total current distribution of the (**a**) DT-MOSFET, (**b**) DS-MOSFET, and (**c**) SPDT-MOSFET.

Figure 5 illustrates the total current distribution of the SPDT-MOSFET, PDT-MOSFET, and DT-MOSFET at *V_GS_* = 15 V and *V_DS_* = 10 V. The introduction of the split-gate sacrifices the conductive path at the bottom of the device’s gate, resulting in a significant reduction in the conduction path and deteriorated on-state characteristics compared to the DT-MOSFET. Apparently, the proposed SiC MOSFET, by introducing the “一”-shaped PSR and an *N_CSL_* layer, greatly increases the current flow path and reducing the *R_on_* of the device.

During the reverse conduction state, the body diode remains in the conduction state and the current flows from the source to the drain through the P-i-N diode. Due to the wide bandgap of SiC, the Von of the P-i-N diode is relatively high. This leads to a rise in *R_on_* and the emergence of the bipolar degradation phenomenon, which results in an amplification of the switching loss [27]. The utilization of lateral integration of the SBD within the sidewalls enhances the performance of the SPDT-MOSFET. We address this issue by integrating the SBD on the sidewall of the SPDT-MOSFET.

**Figure 6:**
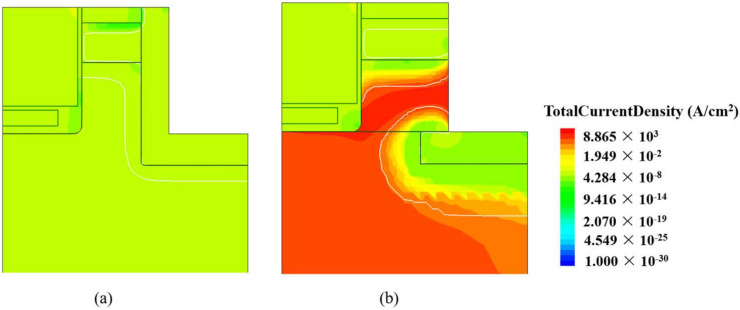

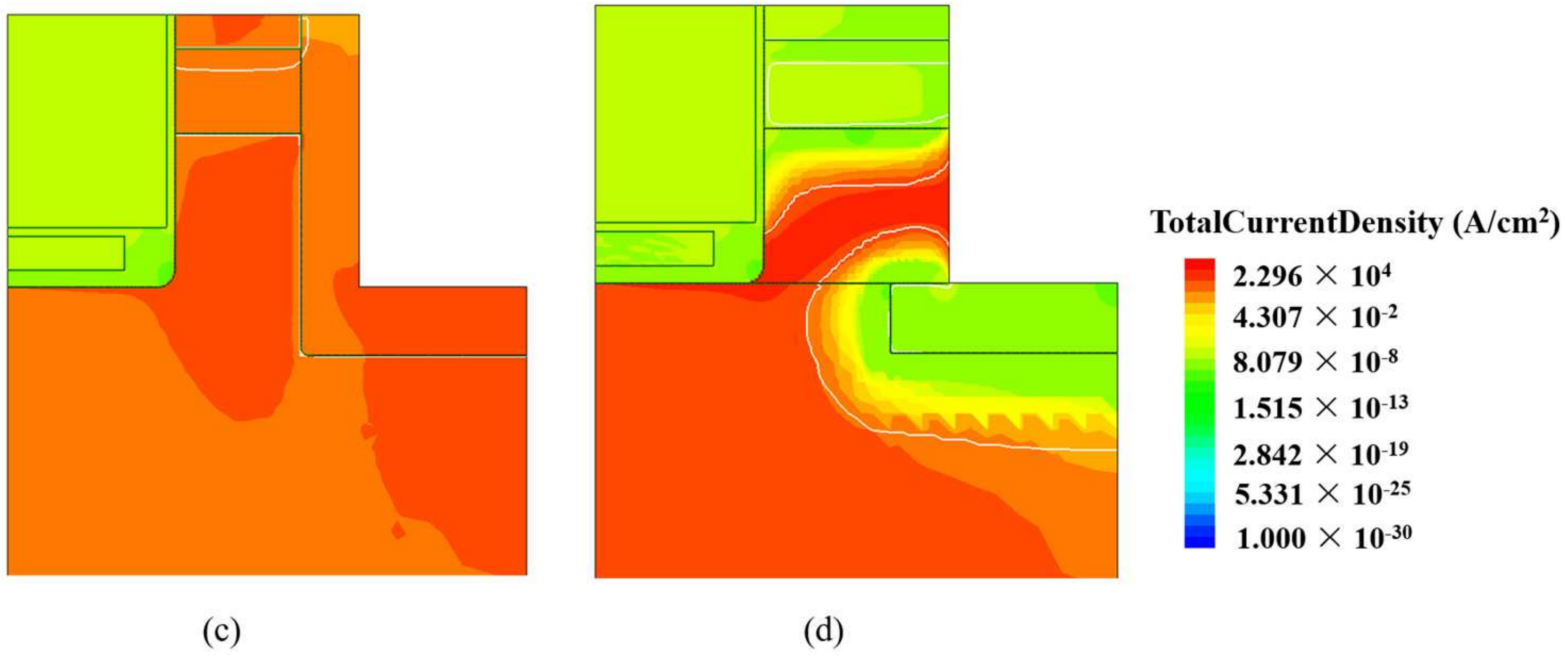
Current distribution of the (**a**) DS-MOSFET at *V_SD_* = 2 V, (**b**) SPDT-MOSFET at *V_SD_* = 2 V, (**c**) DS-MOSFET at *V_SD_* = 3 V, and (**d**) SPDT-MOSFET at *V_SD_* = 3 V.

The current density distributions in the reverse conduction state of the SPDT-MOSFET and DT-MOSFET are illustrated in Figure 6. Apparently, in the proposed SiC MOSFETs, the parasitic body P-i-N diode is inactivated. The reverse current in the SPDT-MOSFET is handled by the SBD. The integrated Schottky diode is located between the P-base region and the PSR layer, which avoids the scenario of an excessively high electric field at the Schottky junction interface, while in the proposed SiC MOSFET, it is the SBD that conducts the reverse current. Therefore, the resistance from the SBD to the P+ shield region in the proposed SiC MOSFET is much lower than the resistance from the N-source to the P+ shield region in the SPDT-MOSFET, which is more conducive to inactivating the parasitic body P-i-N diode.

**Figure 7:**
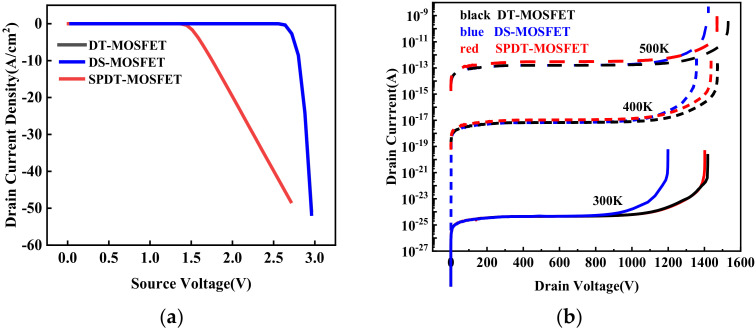
(**a**) Reverse conduction I–V characteristics for the three devices and (**b**) blocking characteristics for the three devices under different temperature conditions.

Figure 7a demonstrates a comparative analysis of the body diode characteristics between the SPDT-MOSFET and DT-MOSFET devices. The DT-MOSFET device exhibits a *V_ON_* of 2.6 V, whereas the SPDT-MOSFET device has a significantly lower *V_ON_* of only 1.5 V. This substantial reduction in *V_ON_*, amounting to a 42.3% decrease, is achieved by integrating an SBD, which effectively suppresses the activation of the body diode. As a result, the SPDT-MOSFET device avoids the phenomenon of bipolar degradation and enhances its reverse conduction capability.

Figure 7b compares the blocking characteristics of the three device structures under different temperature conditions. The breakdown voltages for the proposed structure, the DS-MOSFET, and the conventional SiC DT-MOSFET are 1430 V, 1201 V, and 1437 V at room temperature, respectively. When subjected to reverse voltage stress, the withstand voltage region primarily comprises the P-base and the PSR coupled with the depletion region within the drift region. However, the “一”-shaped PSR and the L-shaped source trench have similar functions in modulating the electric field, effectively protecting the gate oxide and improving the breakdown characteristics of the device.

The leakage current of the three device structures—the proposed DS-MOSFET, the conventional SiC DT-MOSFET, and the SPDT-MOSFET—remains at the same level at room temperature. As the temperature continues to rise, the leakage current of all three devices increases. The inclusion of the SBD in the SiC MOSFET appears to exacerbate the temperature-dependent leakage current issue, but due to the dual protection of the PSR and p-base in the SPDT-MOSFET, this issue has been effectively mitigated [28].

In studies of SiC MOSFET dynamic characteristics, the switching power loss is an important metric for evaluating the switching performance of the devices. Due to the presence of parasitic internal device capacitances, a switching delay occurs during the dynamic switching processes of the devices [29]. This gives rise to conditions where large voltages and currents coexist, leading to increased dynamic power loss. Moreover, the parasitic gate-drain capacitance is a key factor influencing the devices’ switching speeds. Reducing this parasitic capacitance can potentially reduce dynamic power loss by enhancing switching speeds during transitory conditions in SiC MOSFETs.

**Figure 8:**
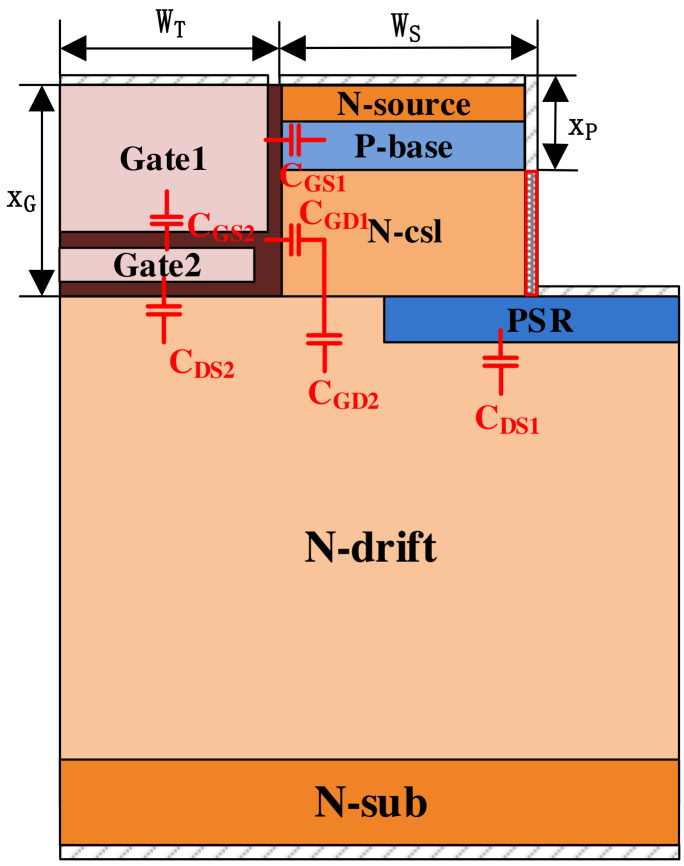
Unit cell cross-sectional view of the SPDT-MOSFET with the capacitances shown.

Figure 8 displays a schematic diagram of capacitances within the SPDT-MOSFET device. The gate-drain capacitance (*C_GD_*) is principally composed of the serial connection between the gate oxide layer capacitance (*C_GD_*_1_) and the drift region depletion layer capacitance (*C_GD_*_2_), as expressed in Equations (1) and (2), respectively:

(1) $$C_{GD1}=\frac{x_{G}-x_{P}}{W_{T}+W_{S}}.\frac{\varepsilon_{OX}}{t_{OX}}$$

(2) $$C_{GD2}=\frac{x_{G}-x_{P}}{W_{T}+W_{S}}.\frac{\varepsilon_{SiC}}{w_{D}}$$

(3) $$C_{GD}=\frac{C_{GD1}.C_{GD2}}{C_{GD1}+C_{GD2}}$$

where $x_{G}$ is the trench gate depth, $x_{P}$ is the P-base region depth, *W_T_* is the trench gate width, *W_S_* is the N-source region width, $\varepsilon_{OX}$ is the dielectric constant of silicon dioxide, $t_{OX}$ is the gate oxide thickness, $\varepsilon_{SiC}$ is the dielectric constant of silicon carbide, and *W_D_* is the N-drift region depth.

**Figure 9:**
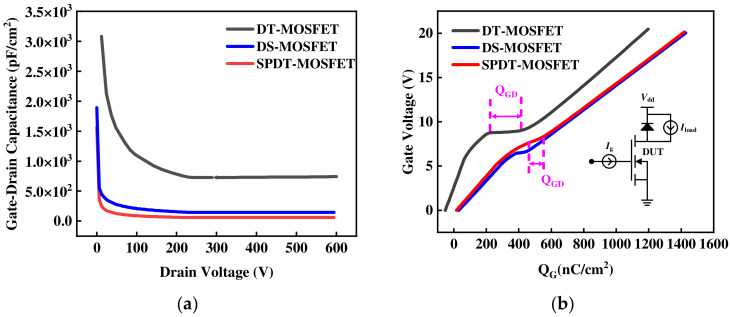
(**a**) Gate-drain capacitance for the three devices and (**b**) gate charge characteristics for the three devices.

Figure 9a shows that, compared to the DT-MOSFET, both the SPDT-MOSFET and DS-MOSFET exhibit a significant reduction in gate-drain capacitance. Specifically, the *C_GD_* values of the SPDT-MOSFET and DT-MOSFET are 140 pF/cm^2^ and 746 pF/cm^2^, respectively, representing a comparative reduction of 81.2%. The introduction of the shielding gate transforms the *C_GD_* located at the bottom of the gate electrode into *C_GS_*. Moreover, as the width of the shielding gate increases, there is a corresponding reduction in *C_GD_*.

Meanwhile, the gate charges were tested using the circuit in the inset of Figure 9b. The load voltage and load current used in the simulation are 100 V DC voltage and 10 A, respectively. The SPDT-MOSFET exhibits a narrower Miller platform and a lower *Q_GD_* value of 115 nC/cm^2^ than that of the DT-MOSFET (195 nC/cm^2^), resulting in a reduction of 41.2%, comparatively, as shown in Figure 9b. The upward gradient of *V_G_* for the SPDT-MOSFET is a little bit lower before reaching the Miller platform because the split-gate shorted to the source contact leads to a portion of *Q_GD_* being transformed into *Q_GS_*. Therefore, the SPDT-MOSFET has a desirable smaller ratio of *Q_GD_* relative to *Q_GS_*. This feature is crucial for suppressing additional losses caused by parasitic parameters in half-bridge circuits, thereby reducing switching losses.

**Figure 10:**
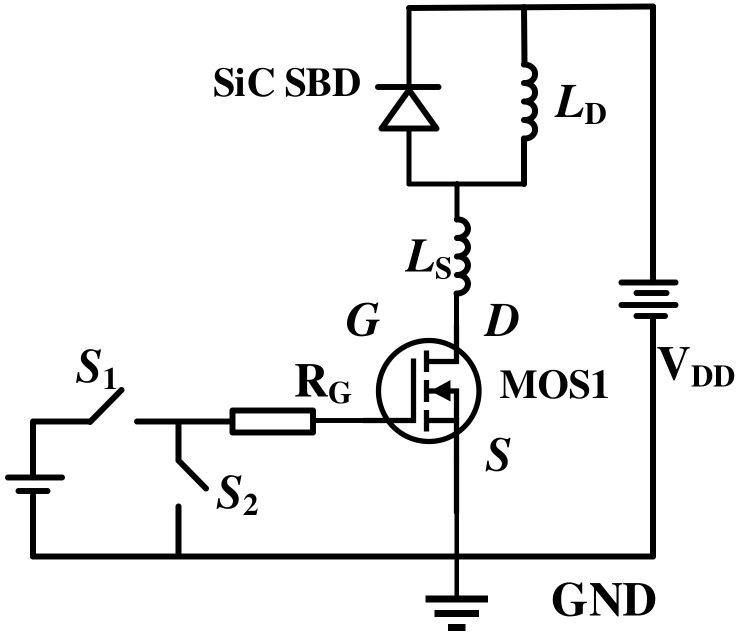
Test circuit for switching characteristics.

The switching performance of the SiC MOSFETs is studied using the test circuit in Figure 10. Within this configuration, MOS1 denotes the device undergoing evaluation, whilst the SiC SBD functions as a reverse freewheeling diode. The supply voltage is *V_DD_* = 600 V. The load inductor is *L_S_* = 200 µH. The gate voltage is ±15 V pulses to set the device to the OFF- and ON-states, respectively.

**Figure 11:**
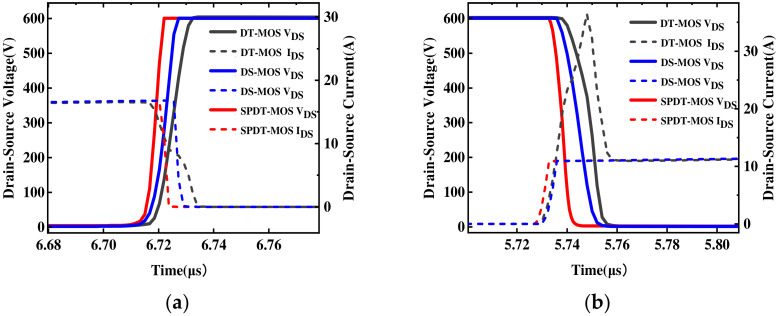
(**a**) Turn-on waveforms and (**b**) turn-on waveforms of the SPDT-MOSFET and DT-MOSFET.

Figure 11 shows the switching waveforms of the proposed structure and the conventional SiC DT-MOSFET. From the graph, it is evident that the SPDT-MOSFET exhibits larger dV/dt compared to the DT-MOSFET. Because of the low *C_GD_* in the SPDT-MOSFET, its switching speed is faster than that of DT-MOSFET [30]. Therefore, due to the smaller gate-drain charge, the SPDT-MOSFET allows for larger dV/dt and lower turn-on loss. The SPDT-MOSFET also has a dip in *I_DS_* while *V_DS_* increases during turn-off, which is caused by capacitive discharge of the freewheeling SBD. Therefore, the turn-on loss and turn-off loss of the proposed MOSFET can be reduced by 35.4% and 40.8% compared to that of the conventional device, respectively.

**Figure 12:**
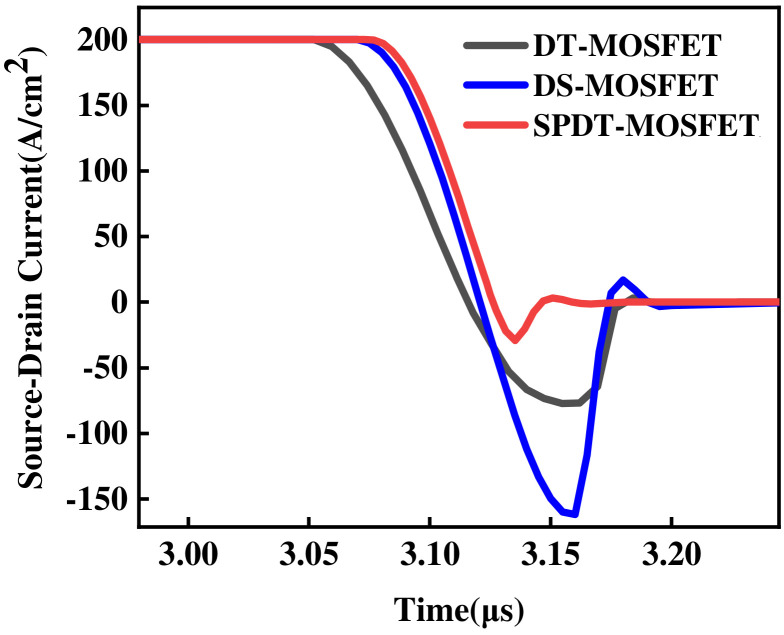
Reverse recovery characteristic comparison for the three devices.

Figure 12 shows the reverse recovery characteristics of the SPDT-MOSFET and the DT-MOSFET. Compared with that of the DT-MOSFET, the reverse recovery peak current (*I_rm_*) of the SPDT-MOSFET is reduced by about 62.05%, and the reverse recovery time (*t_rr_*) of the SPDT-MOSFET is decreased by 34.36% with a value of 507 ns. And the reverse recovery charge (*Q_rr_*) of the DT-MOSFET is 332.45 nC/cm^2^, while that of the SPDT-MOSFET is only 29.85 nC/cm^2^ with a reduction of more than 90.71%. This is because when the SPDT-MOSFET operating in the third quadrant, the integrated SBD effectively impede minority carrier injection into the n-drift region, thus reducing the recombination and minority carrier storage effect during the reverse recovery process. Therefore, the SPDT-MOSFET shows much better reverse recovery performance and greatly reduces the reverse recovery loss.

**Figure 13:**
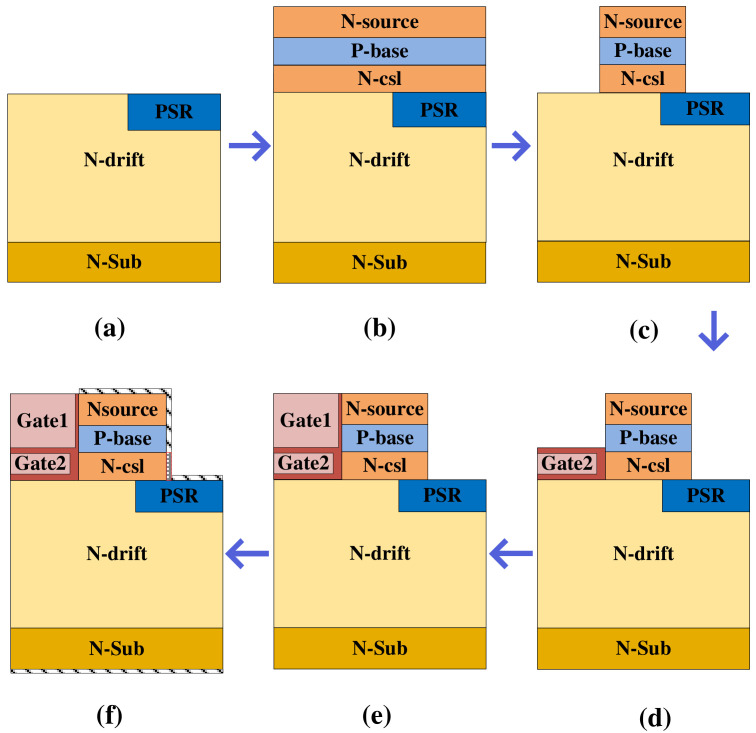
Process flow for fabricating the proposed MOSFET. (**a**) PSR implantation. (**b**) N-csl, P-base, and N+ epitaxial growth. (**c**) Mesa etches. (**d**) Gate 2 oxidation and polysilicon gate deposition. (**e**) Gate 1 oxidation and polysilicon gate deposition. (**f**) Metallization.

Regarding the feasibility of the proposed MOSFET, one potential fabrication process is provided, as shown in Figure 13. First, the PSR is formed by ion implantation [see Figure 13a]. Then, the N-csl, P-base, and N-source regions are sequentially formed through epitaxial growth [see Figure 13b]. Trenches are formed on both sides of the device [see Figure 13c]. Gate oxidation, polysilicon deposition, and polysilicon etch-back are performed [see Figure 13d]. After forming Gate 2, the deposition of polysilicon is performed to form Gate 1 [see Figure 13e]. The final steps are the development of ohmic contacts, SBD contacts, and metallization [see Figure 13f]. This is the only challenging manufacturing process step of the proposed SiC MOSFET, and the formation of SBD metal is crucial.

Table 2 summarizes the performance comparisons between the SPDT-MOSFET, DS-MOSFET, and DT-MOSFET. The SPDT-MOSFET exhibits the expected performance owing to the “一”-shaped PSR and the integrated SBD. 

## 4. Conclusions

A novel SBD-integrated 4H-SiC SGT MOSFET with a “一”-shaped PSR is proposed and studied numerically. The SPDT-MOSFET introduces the “一”-shaped P+ shielding region, which reduces the on-resistance and effectively lowers the surface electric field in the Schottky metal. The simulation results show that the *V_ON_* of the SPDT-MOSFET is 42.3% lower than that of the DT-MOSFET. The *Q_rr_* of the SPDT-MOSFET is 90.7% lower than that of the DT-MOSFET. The total switching losses of the SPDT-MOSFET are 38.1% lower than that of the DT-MOSFET. The above advantages make the SPDT-MOSFET an excellent choice for power electronic applications.

## Figures and Tables

**Table 1:** Simulation parameters for the three MOSFETs.

Parameters	SPDT-MOSFET	DS-MOSFET	DT-MOSFET
Gate oxide thickness (nm)	50	50	50
Schottky contact length (μm)	0.9	-	-
Gate length (um)	1.6	1.6	1.6
P-type Stop Region doping (cm^−3^)	2 × 10^18^	2 × 10^18^	2 × 10^18^
Thickness of split gate (μm)	0.2	0.2	0.2
N-drift epitaxy doping (cm^−3^)	8 × 10^15^	8 × 10^15^	8 × 10^15^
N-drift epitaxy thickness (µm)	11	11	11
Width of half cell (µm)	3.1	3.1	3.1

**Table 2:** Comparison of the simulation results for the three MOSFETs.

Parameters	Device Type		
	SPDT-MOSFET	DS-MOSFET	DT-MOSFET
*V_ON_* (V)	1.5	2.6	2.6
BD	No	Yes	Yes
*V_BR_*^2^/*R_on-sp_* * (GW/cm^2^)	0.85	0.42	0.74
$E_{ox,max}$(MV/cm)	1.5	4.8	4.28
$Q_{GD}\;$(nC/cm^2^)	115	163	195
$C_{gd\;}$(pF/cm^2^) (@V_DS_ = 600 V)	140	144	746
HF-FOM (mΩ·nC)	276	684.6	546
$E_{on}/E_{off}$(mJ/cm^2^)	4.68/0.25	4.61/1.40	7.1/0.42
$Q_{rr}$(nC/cm^2^)	29.85	501.79	332.45

*: *V_GS_* = 15 V, *V_DS_* = 50 V. BD: bipolar degradation.

## Data Availability

Data are contained within the article.
